# Effect of Different Reinforcing Fibers on the Properties of Phenolic Aerogel Composites

**DOI:** 10.3390/gels12020177

**Published:** 2026-02-19

**Authors:** Junjie Xu, Xudong Shao, Lijun Lei, Xin Zhang, Jianlong Chang, Hui Gao

**Affiliations:** 1 School of Mechanical and Electrical Engineering, North University of China, Taiyuan 030051, China; 2 CAS Space Technology Co., Ltd., Beijing 100176, China; 3 School of Energy and Power Engineering, North University of China, Taiyuan 030051, China; 4 School of Materials Science and Engineering, North University of China, Taiyuan 030051, China

**Keywords:** reinforcing fibers, nanoporous structure, ablation resistance, thermal–mechanical–ablative performance

## Abstract

With the rapid development of aerospace technology towards hypersonic vehicles, the synergistic demand for lightweighting and high-efficiency thermal insulation performance of ablation-resistant thermal insulation materials is becoming increasingly urgent. In this study, nanoporous phenolic resin was used as the matrix to prepare quartz fiber-reinforced phenolic aerogel composites (QF/PF), mullite fiber-reinforced phenolic aerogel composites (MF/PF), and carbon fiber-reinforced phenolic aerogel composites (CF/PF), and the influence mechanisms of different reinforcing fibers on the properties of the composites were systematically investigated. QF/PF exhibits optimal thermal insulation performance with a thermal conductivity of 0.1 W/(m·K) at 20–200 °C, followed by MF/PF with a thermal conductivity of 0.11 W/(m·K). Relatively weak thermal insulation performance is demonstrated in CF/PF, whose thermal conductivity reaches 0.14 W/(m·K). However, in terms of mechanical properties, CF/PF is outstanding, with a tensile strength of 54.62 MPa and a bending strength of 29.69 MPa. In addition, the most excellent ablation resistance is displayed in CF/PF, with a linear ablation rate of 0.13 mm/s and a mass ablation rate of 0.0435 g/s, which are significantly lower than QF/PF and MF/PF. This study provides an important basis for the selection of reinforcing fibers in different application scenarios. QF/PF or MF/PF is preferred for high thermal insulation requirements. CF/PF is favored for high load-bearing requirements or extreme ablative environments.

## 1. Introduction

With the rapid development of aerospace technology, aircraft are making continuous breakthroughs toward higher hypersonic speeds and longer endurance, facing increasingly severe aerodynamic thermal environments [1,2]. As a key system for the safe operation of aircraft in extreme thermal environments, the success or failure of a mission is directly determined by the performance of the thermal protection system (TPS) [3,4,5]. During highly maneuverable flight, modern hypersonic vehicles expose their thermal protection materials to extreme conditions exceeding 2000 °C and intense aerodynamic heat flux. Exceptional ablation resistance, thermal insulation properties, and structural load-bearing capacity must be simultaneously demonstrated in these materials [6,7,8]. Therefore, how to break through the bottleneck of existing technology and develop thermal protection materials with superior comprehensive performance stands as an urgent problem in the field of aerospace materials [9,10].

Thermal protection materials in widespread use today are divided into two main categories: ablative and non-ablative. Among them, ablative thermal protection materials, ideal for extreme high-temperature environments, effectively consume heat through physicochemical reactions such as melting, sublimation, and decomposition [11,12,13]. Among many ablative materials, phenolic aerogel composites are widely used in high-temperature components such as rocket nozzles and re-entry vehicles. This is attributable to their high residual carbon rate, excellent thermal insulation properties, and low preparation cost [14,15,16]. As hypersonic vehicles continue to develop towards higher Mach numbers and greater maneuverability, the need for lightweight thermal protection systems is becoming increasingly urgent. In recent years, phenolic aerogel composites have exhibited significant advantages due to their three-dimensional nanoporous structure [17,18,19,20,21]. Phenolic aerogel composites achieve a lightweight effect, with density being reduced to 0.89–1.04 g/cm^3^, which is notably lower than that of traditional phenolic resin composites (1.4–1.8 g/cm^3^). Secondly, exceptional thermal insulation performance is exhibited, and the thermal conductivity can be controlled at 0.079–0.115 W/(m·K) via the continuously distributed nanoporous structure [22]. However, new challenges are also brought in this porous structure. The interfacial bonding strength between the fibers and the resin matrix is weakened with the presence of micropores, which in turn affects the overall mechanical properties of the composites [23]. This issue has become a key bottleneck limiting the application of such materials in hypersonic aircraft. It is worth noting that current research on phenolic aerogel composites mainly focuses on optimizing the porous structure to improve lightweight and thermal insulation performance, yet little attention is paid to the inherent contradiction between nanopore-induced lightweight/thermal insulation performance and mechanical property degradation. More importantly, relevant research still lacks systematic studies on how different types of reinforcing fibers regulate the mechanical, thermal and ablation properties of phenolic aerogel composites. In particular, given the unique nanoporous structure of phenolic aerogels, the influence mechanisms of fiber types on interfacial bonding, stress transfer and ceramic phase formation during ablation remain unclear. Accordingly, the design and application of high-performance nanoporous phenolic aerogel composites have been severely restricted. It has been established through research that the selection of reinforcing fibers is a critical factor in achieving a balance between material lightweighting, ablation resistance performance, and mechanical strength [14,24]. Currently, commonly used fiber reinforcements in the field of extreme thermal protection for hypersonic vehicles mainly include quartz fibers, mullite fibers, carbon fibers, aramid fibers, etc. [25,26,27,28,29]. For example, although the mechanical properties of the material can be significantly improved with carbon fibers, the thermal insulation effect may be reduced to a certain extent [30]. On the other hand, thermal insulation is able to be enhanced with quartz fibers and mullite fibers, yet the requirements of high-strength loads are difficult to meet [31,32]. Most existing studies focus on the performance investigation of phenolic aerogel composites reinforced by a single type of fiber. However, systematic and in-depth analyses regarding the influence laws of different types of reinforcing fibers on the mechanical properties, thermal properties, and ablation properties of phenolic aerogel composites, as well as the material’s micromorphological characteristics and the formation mechanism of ceramic phases during the ablation process, are still lacking in relevant research at present. To address the core contradiction of nanoporous phenolic aerogel composites, a comparative investigation on the application of three typical reinforcing fibers (quartz fibers, mullite fibers, carbon fibers) in the nanoporous phenolic resin matrix is innovatively performed in this study. Different from previous studies focusing on single fibers, the regulation laws of fiber types on the comprehensive performance of phenolic aerogel composites are systematically revealed in this work. In addition, the coupling mechanism between fiber chemical composition/skeleton structure and the performance of the nanoporous matrix is clarified, and a targeted solution is provided to solve the contradiction between lightweight/thermal insulation performance and mechanical properties of nanoporous phenolic aerogel composites. Therefore, the influence mechanism of different kinds of fibers on the comprehensive performance of nanoporous phenolic aerogel composites is systematically studied. On one hand, such research helps deepen understanding of the synergistic interaction between fibers and porous matrices. On the other hand, it provides theoretical foundation and practical guidance for the optimized design of a new generation of lightweight thermal protection materials.

In this study, phenolic aerogel composites reinforced with quartz fibers, mullite fibers and carbon fibers are prepared using the resin transfer molding (RTM) process. Using organosilicon-modified phenolic resin as the matrix [33,34], a continuous nanoporous structure is constructed via the sol–gel and ambient pressure drying method and combined with the ceramic cladding layer formed by ceramizable materials after ablation, thus achieving the synergistic improvement of the material’s ablation resistance and thermal insulation effect. Systematic mechanical property tests (including tensile and bending tests) and oxygen–acetylene ablation tests are conducted. The focus of these tests is to explore how different fiber types influence the mechanical properties, interfacial bonding characteristics, and ablation resistant behaviors of the composites. By combining micro-morphological and compositional analyses, the correlation between fiber types and material properties is elucidated in depth. Such an in-depth elucidation provides an experimental basis for the design and optimization of lightweight and high-performance thermal protection materials.

## 2. Results and Discussion

### 2.1. Microscopic Morphology and Physical Properties

**Figure 1:**
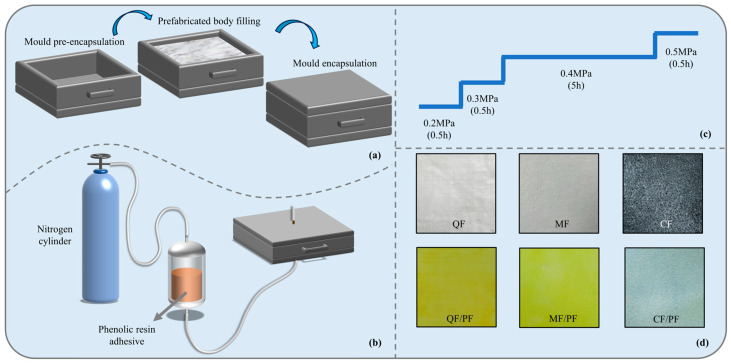
Schematic diagram of the composite preparation and the morphology of the three fiber knitted felts before and after preparation. (**a**) Knitted felts loaded into the mold. (**b**) Resin injection. (**c**) Four-stage pressure injection process. (**d**) Forms of the three types of knitted felts before and after preparation.

A schematic of the preparation of the composites and the morphology of the three fiber knitted felts before and after preparation are shown in Figure 1. Among others, the operation of loading the knitted felts into the molds and injecting the resin into the molds by means of high-temperature pressurization is depicted in Figure 1a,b. The injection process adopts a four-stage pressure control strategy, as illustrated Figure 1c. The initial stage (0.5 h): Maintains a low pressure of 0.2 MPa to ensure the uniform advancement of the glue front. The first and middle stage (0.5 h): Maintains a pressure of 0.3 MPa to further enhance the flow and infiltration speed of the glue. The middle stage (5 h): Raises the pressure to 0.4 MPa to promote the complete infiltration of the knitting felt. The later stage (0.5 h): Increases the pressure to 0.5 MPa to compensate for shrinkage. The macroscopic morphology of the knitted felts with the three fibers is presented in Figure 1d. The cured composites are all beige in color due to the similar color of the quartz and mullite fiber precasts. Conversely, the carbon fiber preform is black, and its cured composite is close to dark green in color. To facilitate subsequent analysis, the three composites were assigned distinct designations: quartz fiber-reinforced phenolic aerogel composite (QF/PF), carbon fiber-reinforced counterpart (CF/PF), and the mullite fiber-reinforced one (MF/PF).

**Figure 2:**
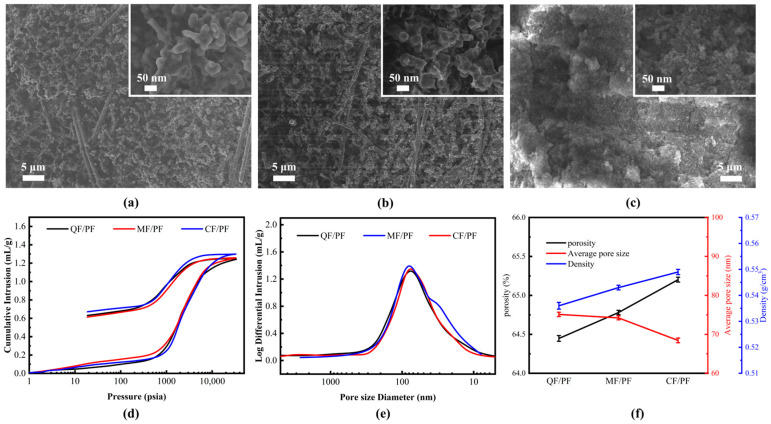
Microscopic morphology and porosity of the resin matrix. (**a**–**c**) QF/PF, MF/PF, and CF/PF, respectively. (**d**) Mercury injection-ejection curve. (**e**) Pore size distribution curve. (**f**) Average pore size, porosity and density.

The microscopic morphology of the resin matrix of three different fiber-reinforced composites is compared in Figure 2. Despite the differences in the materials of the reinforcing fibers, the overall morphological characteristics of the resin matrix display a high degree of consistency. The interlinked and tightly stacked resin microcluster structure is all formed from cured nanoporous phenolic resin [35]. This structural feature effectively extends the solid-phase heat transfer path and enhances the material’s thermal insulation performance. Meanwhile, it can be observed that obvious nanoscale pores exist in the interfacial region between the resin matrix and the reinforcing fibers. The nanoporous structure at the interface may reduce the efficiency of stress transfer, which in turn affects the mechanical properties of the composites. It is particularly noteworthy that the resin matrix exhibits a slight local aggregation phenomenon in CF/PF. The phenomenon manifests as enrichment of resin microclusters on the fiber surface, causing partial disappearance of the nanoporous structure. Meanwhile, the fibers are tightly wrapped in the excess resin matrix, forming a dense “resin-fiber” composite unit.

The pore structure characteristics of the three different fiber-reinforced composites are analyzed through mercury injection testing. A clear hysteresis loop is evident in the mercury injection and ejection curves. There is an implication that the internal pores of the material have good connectivity, which is beneficial for the thermal protection material to achieve effective thermal buffering during the ablation process. From the mercury injection curve, it can be seen that Hg injection grows slowly in the low-pressure stage (<1000 Psia), indicating low content of micrometer-sized macropores in the material. When the pressure reaches 1000 Psia, a sharp upward trend is exhibited in the curve, indicating that a highly homogeneous pore structure is formed inside the material. It is worth noting that the mercury injection–ejection curves and the pore size distribution curves of the three composites almost completely overlap. The overlap suggests that the final pore structure characteristics of the composites are less influenced by the different types of fiber reinforcement. The pore structure characteristics of the three fiber-reinforced composites are highly similar: porosities are stabilized at approximately 65%, and densities are stabilized at around 0.54 g/cm^3^. The average pore size of both QF/PF and MF/PF is around 75 nm, while the average pore size of CF/PF is slightly smaller to 68.44 nm. This highly controllable pore structure ensures that the materials possess excellent thermal insulation properties, highlighting the dominant role of the resin matrix in pore formation.

**Figure 3:**
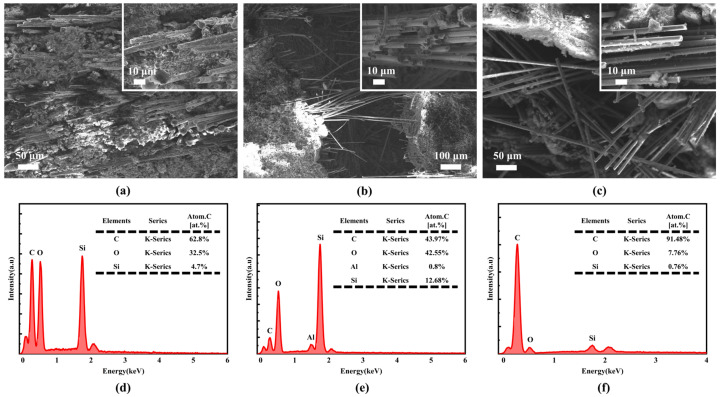
Fiber micro-morphology and elemental analysis. (**a**–**c**) QF/PF, MF/PF and CF/PF micro-morphology, respectively. (**d**–**f**) Elemental analyses of QF/PF, MF/PF and CF/PF, respectively.

The micro-morphology and elemental distribution characteristics of the fracture sections of the three fiber-reinforced composites are presented in Figure 3. Micro-morphology observations reveal that some fibers are pulled out during the fracture process yet retain their intact morphology. The fibers are confirmed to play an effective reinforcing role in the composites, which further suggests that they possess excellent mechanical integrity themselves. Meanwhile, it is found that the surface of the unextracted fibers is basically uniformly covered by the resin, and the interfacial bonding is tight. No obvious defects such as interfacial debonding or cracks are observed. It is shown through energy spectrum analysis results that the three characteristic elements (C, O, and Si) are all contained in the fibers of the three composites. However, significant differences are present in their relative contents. In CF/PF, the content of elemental C is as high as 91.48 at%, which is much higher than that in QF/PF (62.8 at%) and MF/PF (43.97 at%). This finding is consistent with the high carbon properties of carbon fibers. The characteristic Al element is detected in MF/PF. However, the fiber surface is covered with the resin matrix, leading to a relatively low detection amount of the Al element.

### 2.2. Thermal Properties

**Figure 4:**
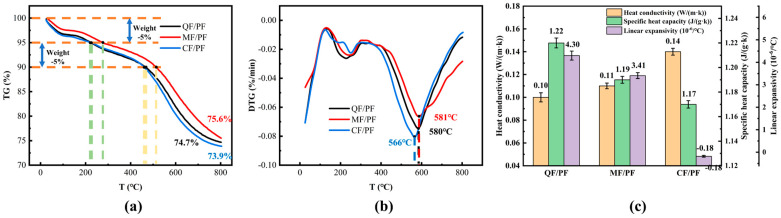
Thermal properties of composites. (**a**) TG, (**b**) DTG, (**c**) Thermal conductivity, specific heat capacity and coefficient of linear expansion.

The thermal properties of the three fiber-reinforced composites are compared in Figure 4. TGA reveals that the three materials exhibit similar thermal weight loss trends, mainly due to their identical phenolic resin matrix. It is also found that the thermogravimetric process displays a typical three-stage feature. The initial stage (0~300 °C) is dominated by weight loss from two sources: volatilization of residual solvents (e.g., ethanol) and escape of low-molecular-weight linear polymers, as well as dehydration, aromatization, and preliminary carbonation of resin molecules. When the temperature rises to 300–580 °C, the methylene bond between the molecular chains of the resin starts to break rapidly. In this process, small-molecule gases such as CH_4_, CO_2_, and H_2_O are released [36], accompanied by the deep carbonization of the resin matrix, with the weight loss rate peaking at this stage. At the high-temperature stage of 580–800 °C, the resin molecular chains are further dehydrated and condensed. However, the decomposition rate gradually slows down, and a stable porous carbon skeleton structure is eventually formed. It is noteworthy that relatively low thermogravimetry is exhibited in MF/PF throughout the temperature interval. Up to 470 °C, the thermogravimetric curves of QF/PF and CF/PF almost overlap. The fastest rate of thermogravimetry is demonstrated in all three materials in the 500–700 °C interval. The fastest decomposition temperature of CF/PF (566 °C) is significantly lower than that of QF/PF (580 °C) and MF/PF (581 °C). This difference may be related to the fact that the higher thermal conductivity of the carbon fibers facilitates heat transfer, thus accelerating the decomposition of the resin. At 800 °C, it is shown that the residual carbon amounts of the three materials are very similar: 74.7% for QF/PF, 75.6% for MF/PF, and 73.9% for CF/PF. It is proven that even though different types of reinforcing fibers are used in the composites, small differences in residual carbon rates are resulted from the use of the same resin matrix. The above results indicate that the type of reinforcing fiber has little effect on the thermal stability of phenolic aerogel composites.

Significant differences in thermal conductivity properties are exhibited in the composites reinforced with quartz fiber, mullite fiber, and carbon fiber. The best thermal insulation performance is demonstrated in QF/PF, with the lowest thermal conductivity among the three materials at 0.10 W/(m·K) and the highest specific heat capacity at 1.22 J/(g·K). The heat transfer rate is effectively slowed down due to low thermal conductivity, and the material is endowed with enhanced heat storage capacity by virtue of its high specific heat capacity. These two factors collectively contribute to a notable reduction in the efficiency of heat transfer into the material. The thermal conductivity of MF/PF is 0.11 W/(m·K), approximately 9.1% higher than that of QF/PF. It is significantly lower than that of CF/PF (0.14 W/(m·K)), representing a 21.4% decrease. When combined with its moderate specific heat capacity, MF/PF is shown to still possess good thermal insulation efficiency. In contrast, relatively low thermal conductivity and specific heat capacity are exhibited in carbon fiber composites, with poor thermal insulation performance resulting therefrom. It is endowed with significantly higher heat conduction efficiency than the other two materials, which are not conducive to heat insulation. In terms of thermal expansion behavior, very different characteristics are demonstrated in the three composites. The unique negative thermal expansion characteristic is displayed in CF/PF, with a coefficient of thermal expansion of −0.18 × 10^−6^/°C. Alternatively, positive thermal expansion characteristics are manifested in QF/PF and MF/PF, with their coefficients of thermal expansion being 4.3 × 10^−6^/°C and 3.41 × 10^−6^/°C, respectively. This is because the carbon fiber shrinks when heated, and the degree of shrinkage is greater than the thermal expansion of the phenolic resin matrix, resulting in the overall composite showing a negative thermal expansion phenomenon [37,38].

**Figure 5:**
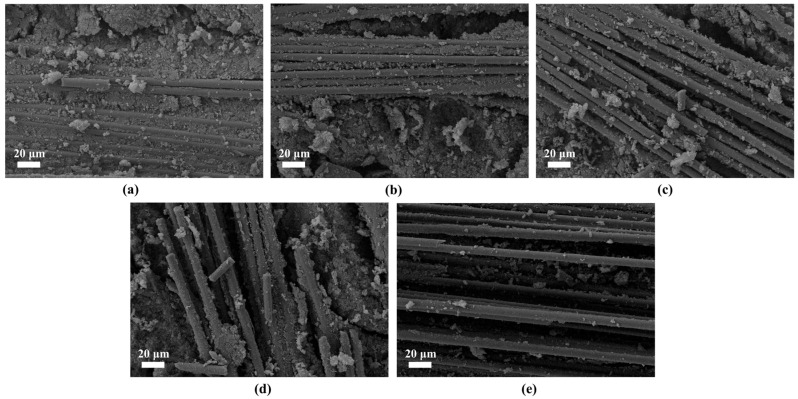
Interfacial bonding status between fibers and the resin matrix under different numbers of thermal expansion coefficient tests. (**a**) Untested. (**b**) After one test. (**c**) After two tests. (**d**) After three tests. (**e**) After four tests.

Owing to the opposite coefficients of thermal expansion between the phenolic resin matrix and carbon fibers in CF/PF, significant thermal stress is generated at the interface of the resin matrix and fibers. When CF/PF undergoes temperature changes, the shrinkage tendency of carbon fibers and the expansion tendency of the resin matrix interact in a tensile manner: the resin matrix expands outward while carbon fibers tend to shrink, and a thermal stress field with alternating tension and compression is easily formed at the interface through this interaction. For this reason, four repeated thermal expansion coefficient tests are conducted on the same CF/PF specimen, and the interfacial bonding state between the resin matrix and fibers in the original state and after the four tests is observed using a scanning electron microscope, as detailed in Figure 5. Excellent bonding performance is exhibited between the phenolic resin matrix and carbon fibers in the original untested material, where the carbon fibers are completely encapsulated by the resin matrix without obvious gaps or defects. After the first thermal expansion coefficient test, obvious separation occurs between the carbon fiber bundles and the resin matrix on both sides, and the interfacial gaps increase significantly. After the second test, the interfacial bonding performance between the resin matrix and fibers among the fiber bundles decreases, leading to the gradual expansion of gaps between the fiber bundles. After the third test, the debonding phenomenon is significant both between the fiber bundles and the phenolic resin matrix; a large amount of the resin matrix on the fiber surface peels off, and cracks also appear in the resin matrix. After the fourth test, it is found that the peeling of the resin matrix between the fiber bundles becomes more significant, and the carbon fibers exhibit clear contours. The above phenomena indicate that repeated thermal cycles induce interfacial damage in CF/PF, which in turn impairs the mechanical properties of the composite. Meanwhile, the expanded interfacial gaps and microcracks will serve as channels for high-temperature gas flow to invade the interior of the material, thereby reducing the ablation resistance of the material. Therefore, this phenomenon should be avoided as much as possible.

### 2.3. Mechanical Properties

**Figure 6:**
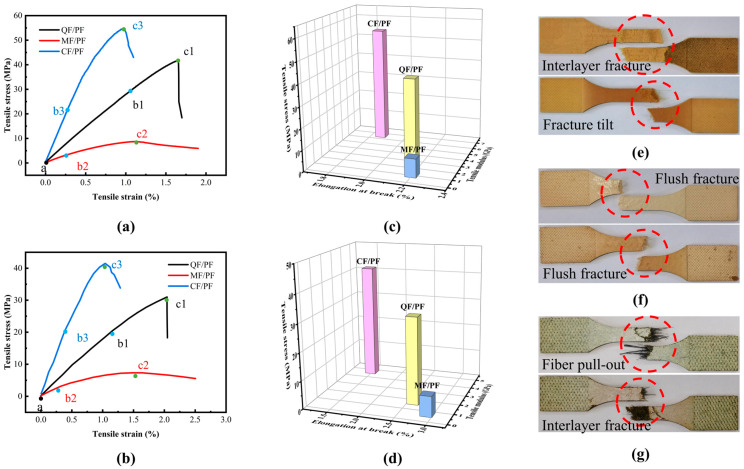
Tensile properties. (**a**) Tensile stress–strain curve at 25 °C. (**b**) Tensile stress–strain curve at 200 °C. (**c**) Tensile strength, elongation at break and tensile modulus at 25 °C. (**d**) Tensile strength, elongation at break and tensile modulus at 200 °C. (**e**–**g**) Fracture morphology of QF/PF, MF/PF and CF/PF.

The tensile properties of the three fiber-reinforced composites at 25 °C and 200 °C are compared in Figure 6, while the tensile stress and tensile modulus at the two temperatures are shown in Table 1 and Table 2, respectively. The test results indicate that CF/PF exhibits the highest tensile strengths at both temperatures (25 °C: 54.62 MPa, 200 °C: 41.6 MPa). This is primarily due to the inherent high strength of carbon fibers themselves. In contrast, the tensile strength of QF/PF is lower (25 °C: 41.66 MPa, 200 °C: 31.14 MPa), and the lowest tensile strength is displayed in MF/PF (25 °C: 8.71 MPa, 200 °C: 7.34 MPa), which is again associated with the relatively low inherent strength of quartz fibers and mullite fibers. At 25 °C, the tensile strength of CF/PF is 31.1% and 527.1% higher than that of QF/PF and MF/PF, respectively. At 200 °C, the tensile strength of CF/PF is 33.6% and 466.8% higher than that of QF/PF and MF/PF, respectively. Meanwhile, different tensile moduli are exhibited by the three composite materials. Among them, the highest tensile modulus at 25 °C and 200 °C is demonstrated in CF/PF, indicating that it has stronger deformation resistance and is more conducive to improving the stability of the structure. It can be seen that the advantage of CF/PF in tensile properties is very prominent whether in both normal and high-temperature environments, and it is the best choice for coping with harsh loading conditions among the three materials.

The fracture process of composites under tensile loading can be divided into three typical stages. First is the elastic stage (a–b), where the stress–strain relationship is linear, only recoverable elastic deformation occurs, and internal defects and microcracks remain unexpanded. As the load increases and the material enters the plastic phase (b–c), internal microcracks in the matrix begin to initiate and interconnect to form macrocracks, while the fiber/resin interface is gradually debonded. The mechanical properties at this stage mainly depend on the strength of the fibers themselves and the bonding properties of the fiber/resin interface. Finally, in the failure stage, after the matrix is completely fractured, the load is primarily borne by the fibers. Notable differences are evident among the different fiber systems. Brittle fracture characteristics, with stress dropping suddenly after fracture, are exhibited in CF/PF and QF/PF. In contrast, progressive failure behavior, with stress decreasing slowly after fracture, is shown in MF/PF. After debonding from the matrix, mullite fibers can continue to carry the load through elastic elongation. The load-bearing capacity is maintained until the fibers reach their ultimate strength and undergo complete fracture. In contrast, carbon and quartz fibers are difficult to sustain load bearing after the matrix fractures, resulting in sudden fracture.

Significant attenuation in tensile properties is evident for all three fiber-reinforced composites at 200 °C compared with that at the 25 °C condition, arising from the initiation of resin matrix pyrolysis under high temperatures. Gases generated during pyrolysis accumulate within the material, resulting in increased internal pressure. This elevated pressure, in turn, leads to the expansion and interconnection of existing defects and cracks, ultimately forming larger cracks. At the same time, the material’s porosity is elevated and the bonding strength between fibers and the resin matrix is weakened due to the increase in internal pressure. These changes ultimately result in a notable decrease in tensile strength.

Through comparing the tensile fracture morphologies of the three fiber-reinforced composites at 25 °C and 200 °C (upper figure: 25 °C, lower figure: 200 °C), marked fracture differences can be discerned. Significant interlaminar fracture characteristics are exhibited in both QF/PF and CF/PF. The phenomenon is mainly attributed to the insufficient interlaminar bond strength of the knitted mat reinforced structure. When the tensile stress reaches a critical value, the interlayer interface is preferentially destroyed. It is noteworthy that CF/PF exhibited pronounced fiber pull-out during the fracture process. Such behavior indicates that carbon fibers possess excellent tensile strength, which is sufficient to resist the bonding force between the resin matrix and the fibers, allowing the fibers to be pulled out. In contrast, the typical flush fracture morphology is evident in MF/PF, suggesting that after the matrix fractures, the mullite fibers cannot resist the bonding force between the resin matrix and the fibers and thus fracture along with the fracture location.

**Figure 7:**
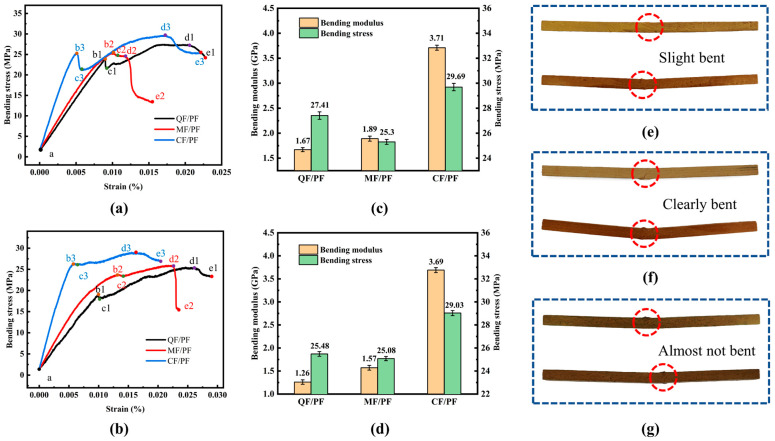
Bending properties. (**a**) Bending stress–strain curve at 25 °C. (**b**) Bending stress–strain curve at 200 °C. (**c**) Bending strength and bending modulus at 25 °C. (**d**) Bending strength and bending modulus at 200 °C. (**e**–**g**) Fracture morphology of QF/PF, MF/PF and CF/PF.

The bending performance curves of the three fiber-reinforced composites at 25 °C and 200 °C are illustrated in Figure 7. A typical four-stage characteristic is shown in the curves. In the initial loading stage (a–b), the material undergoes linear elastic deformation. The stress–strain relationship is in accordance with Hooke’s law, and no obvious damage is produced inside the composite material at this time. When the stress reaches the yield strength at point b, the material enters the plastic deformation stage (b–c). At this stage, microcracks inside the matrix begin to sprout and expand, and the fibers bend and rotate, causing the load-bearing capacity to decrease and the stress to drop obviously. After point c, the material exhibits work-hardening behavior (c–d). This behavior primarily originates from the combined effect of the fibers in the upper half of the specimen being stretched and those in the lower half being compressed. Simultaneously, strain strengthening is contributed to through the plastic flow of the resin matrix on the compression side and the reorientation of the fibers. Eventually, when the stress reaches the ultimate bending strength at point d, the material fails completely due to macroscopic crack penetration.

The bending strength test results of the three fiber-reinforced composites show that CF/PF exhibits superior mechanical properties under both room temperature and high-temperature environments. At room temperature, the bending strength of CF/PF reaches 29.69 MPa, which is 8.32% and 17.35% higher than that of QF/PF (27.41 MPa) and MF/PF (25.3 MPa), respectively, ranking first among the three. When the temperature rises to 200 °C, the bending strength of CF/PF decreases slightly to 29.03 MPa, while that of QF/PF and MF/PF decreases to 25.48 MPa and 25.08 MPa, respectively. Under this high-temperature condition, the bending strength of CF/PF is still 13.93% and 15.75% higher than that of QF/PF and MF/PF, respectively. In conclusion, whether in room temperature or high-temperature environments, superior bending resistance and stronger structural reliability are demonstrated by CF/PF. From the bending performance test results, it is evident that the bending strength and modulus of the three fiber-reinforced composites are higher at 25 °C than under the 200 °C test condition. This trend is consistent with the change rule of tensile properties. The pyrolytic reaction of the resin matrix in the high-temperature environment leads to the rise in internal pressure of the material and the decrease in the bond strength at the fiber/matrix interface. The reduction in mechanical properties is attributed to these factors in combination. It is worth noting that the stress drop of the material after crossing the yield point is smaller at 200 °C than that at 25 °C. Among the three materials, the best ability to maintain high temperature properties is exhibited in CF/PF. At 200 °C, its bending strength and modulus decrease by only 2.22% and 0.54%, respectively, a performance much better than that of QF/PF and MF/PF. It is fully demonstrated that degradation of the resin matrix results from high temperature. However, the weakening of the matrix can be effectively compensated for through the excellent thermal stability and mechanical properties of carbon fibers. The decline in the overall performance of the composites is thereby significantly slowed down.

By comparing the bending fracture morphology of the three fiber-reinforced composites at 25 °C and 200 °C (upper figure: 25 °C, lower figure: 200 °C), marked performance differences can be observed. Obvious bending fracture characteristics are exhibited in MF/PF, with the fracture showing a clear destructive morphology. On the other hand, the least pronounced bending fracture behavior and a flatter fracture surface are demonstrated in CF/PF. Further external bending tests on the fractured specimens revealed that high bending resistance is maintained in CF/PF even after fracture. In contrast, QF/PF and MF/PF are more susceptible to bending deformation after fracture. This difference is mainly due to the higher modulus of elasticity of the carbon fibers, which allows CF/PF to maintain a better structural integrity after fracture [39,40]. It is noteworthy that the fracture morphology at 200 °C shows more obvious plastic deformation characteristics, which is related to the partial pyrolysis of the resin matrix due to high temperature. However, due to their excellent mechanical properties, the best bending resistance is still exhibited in carbon fibers.

**Figure 8:**
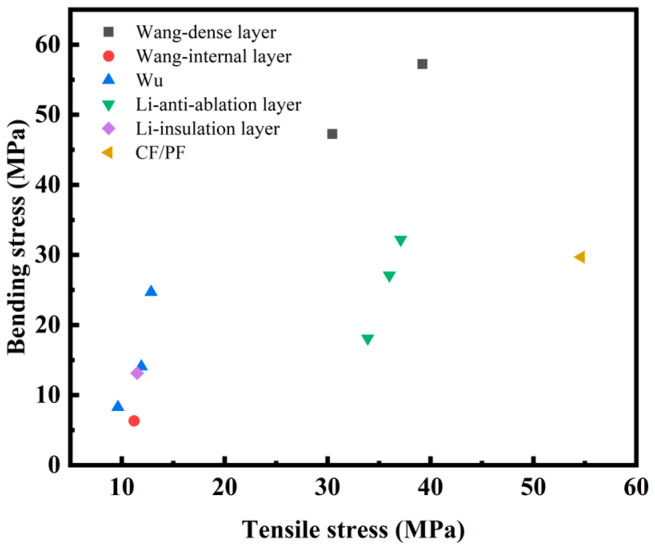
Comparison of mechanical properties between CF/PF and similar composites [41,42,43].

The mechanical advantages of the CF/PF composite with the optimal mechanical properties in this study are clearly demonstrated in Figure 8 through a horizontal comparison of its tensile and bending properties with those of other similar composites. The tensile properties of CF/PF are significantly superior to all the compared materials, exhibiting outstanding tensile load-bearing capacity. In comparison to the phenolic aerogel composite prepared by Wu et al. [41], CF/PF shows clear advantages in both tensile and bending performance. Although its bending properties are slightly lower than those of the dense layer of phenolic aerogel composites developed by Wang et al. [42] and the sample with a fiber content of 55% in the anti-ablation layer developed by Li et al. [43], it still ranks at an excellent level among similar materials. Overall, excellent comprehensive mechanical properties are exhibited by the CF/PF composite, and a significant competitive advantage is demonstrated especially in tensile properties, which provides strong support for its application in scenarios with high load requirements.

### 2.4. Ablative Morphology

**Figure 9:**
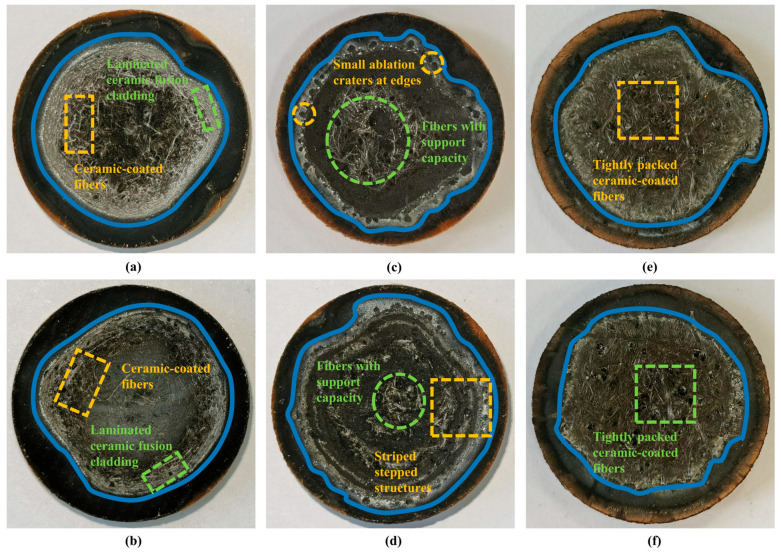
Ablation morphology analysis. (**a**) QF/PF in case1. (**b**) QF/PF in case4. (**c**) MF/PF in case1. (**d**) MF/PF in case4. (**e**) CF/PF in case1. (**f**) CF/PF in case4.

To investigate the ablation properties of the three fiber-reinforced composites under different heat flux densities, four heat flux conditions were employed in the experiment. The ablation rates of each material were measured, and the ablation morphology and elemental composition of the materials were analyzed. The detailed heat flux conditions are listed in Table 3. The ablation morphology of the three fiber-reinforced composites for case1 and case4 is depicted in Figure 9. When the ablation temperature reaches 200 °C, the phenolic resin matrix is pyrolyzed. During this process, gaseous products such as CO, CO_2_ and benzene ring derivatives are released, while the resin matrix gradually carbonizes and shrinks. As the temperature continues to increase to 800 °C, the resin matrix is completely transformed into a porous carbon skeleton structure. During this process, quartz and mullite fibers melt in the high-temperature oxidation environment. Most of the molten material is stripped off under the shear effect of the high-speed gas flow, while the remaining molten material penetrates into the pores of the carbon layer and bridges the fibers and matrix. The composite material’s resistance to physical stripping is enhanced through such penetration. In comparison, more excellent ablation resistance is shown in CF/PF, owing to its higher thermal stability and intact fiber backbone structure.

By comparing and analyzing the ablation morphology of three types of fiber-reinforced composite materials, significant differences can be observed. A large number of silver–white ceramic phases are distributed on the surface of QF/PF. In the middle region of the ablation crater, owing to the strong heat flow scouring effect, the ceramic fused cladding layer is less, exposing the carbon layer organization. On the other hand, a typical layered ceramic cladding structure is formed in the edge region. Fibers encapsulated within the ceramic phase can be seen around the middle region of the ablation pit. It is noteworthy that under higher heat flow conditions (case4), the fused cladding layer is markedly reduced. This is because stable attachment of liquid ceramic tissues on the surface becomes difficult, as a result of the strong scouring effect. MF/PF exhibits unique ablation characteristics: a ceramic phase-encapsulated fiber structure with support capability is generated in the middle of the ablation craters, and small ablation holes are present in the edge regions. Under higher heat flow conditions (case4), a strip-like stepped structure is formed around the ablation crater. Quartz and mullite fibers are effective in carrying away heat through melt absorption at high temperatures. However, a large number of fibers melt, which reduces their support capacity. As a result, the resin matrix is directly exposed to the ablation environment, thus accelerating the overall ablation rate. CF/PF exhibits a very different ablation behavior: under the protection of the surface SiC coating, the carbon fiber skeleton remains intact at high temperatures, with no significant melting observed. The heat absorption effect of melting is lacking in carbon fibers, and their high thermal conductivity is not conducive to thermal insulation. However, the overall ablation rate of the material is substantially reduced through the intact fiber backbone. The difference in the ablation appearance of CF/PF under the two heat flow conditions is relatively small. It is mainly manifested as a change in the density of the surface fiber tissue, and ceramic melt cladding is barely observed on the fiber surface. The property suggests that carbon fibers resist ablation erosion mainly through maintaining the support provided by the intact structure. Although their high thermal conductivity causes a reduction in thermal insulation, a clear advantage in terms of scour resistance is exhibited in them.

**Figure 10:**
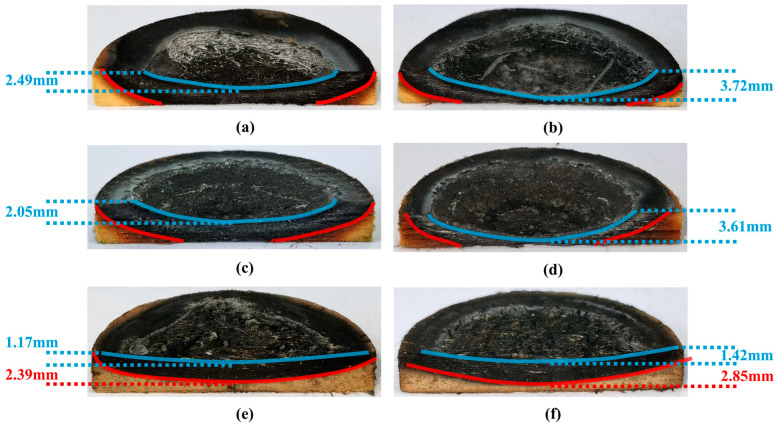
Ablation cross-section. (**a**) QF/PF in case1. (**b**) QF/PF in case4. (**c**) MF/PF in case1. (**d**) MF/PF in case4. (**e**) CF/PF in case1. (**f**) CF/PF in case4.

The ablation cross-section morphology of the three fiber-reinforced composites under different oxyacetylene flow conditions is illustrated in Figure 10. It can be clearly observed from the comparative analysis that the ablation pit depth of QF/PF and MF/PF increases significantly with the increase in the oxyacetylene flow rate (as shown in the blue curve). In contrast, the ablation depth of CF/PF changes relatively gently. The difference is mainly attributed to the high-temperature stability of carbon fibers, which can still maintain an intact skeleton support structure under extreme ablation conditions [44,45]. Further observation of the materials’ cross-sectional characteristics reveals that a denser structure of the carbonized layer (between the blue and red markers) is demonstrated in QF/PF and MF/PF. The densification is mainly attributed to the synergistic effect between the fiber melt phase and the ceramic melt generated during the oxidation of the ceramizable material, which effectively fills the pores in the carbonized layer. In addition, the interfacial bonding between the fibers and the substrate is significantly enhanced through the penetration of the melt into the pyrolysis layer. The enhancement makes the internal structure of the material more stable, resulting in a denser carbonation layer structure. The greatest ablation depth is found in QF/PF, with ablation depths of 2.49 mm and 3.72 mm under case1 and case4, respectively. The second deepest ablation depth is observed in MF/PF, with corresponding ablation depths of 2.05 mm and 3.61 mm under the same conditions. The best ablation resistance is shown in CF/PF, with ablation depths of only 1.17 mm and 1.42 mm. Further comparison of the data indicates that the ablation depth of CF/PF is reduced by 53% and 61.8% compared to QF/PF for case1 and case4, respectively. It can be seen that with the increase in ablation temperature, the attenuation of the ablation resistance of CF/PF is less than that of QF/PF. It is particularly noteworthy that under the same ablation conditions, only CF/PF lacks a carbonized layer throughout their entire sample thickness. This feature visually confirms their superior ablation resistance compared to QF/PF and MF/PF. Such superiority is mainly due to the continuous support provided by the carbon fiber backbone.

**Figure 11:**
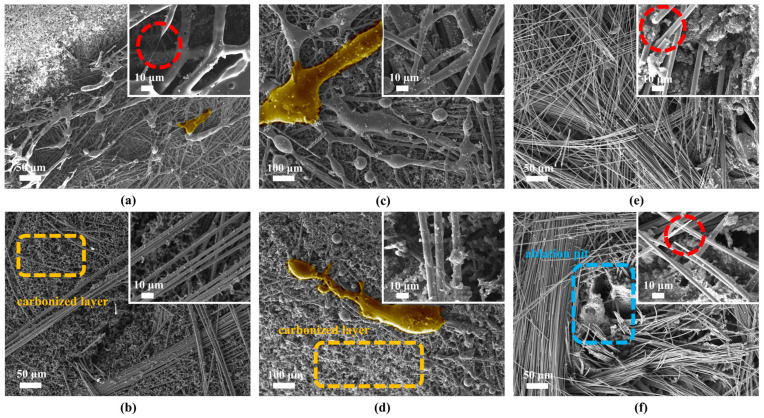
Micro-morphology of ablation. (**a**) QF/PF in case1. (**b**) QF/PF in case4. (**c**) MF/PF in case1. (**d**) MF/PF in case4. (**e**) CF/PF in case1. (**f**) CF/PF in case4.

The ablation micro-morphological characteristics of the three fiber-reinforced composites under different operating conditions are demonstrated in Figure 11. Excellent shape preservation ability is shown in CF/PF under both case1 and case4. The ablated surface is covered with the intact retained carbon fibers. It is noteworthy that the ablated carbon fibers still maintain their original morphological characteristics, with no melting or sublimation observed. Such behavior fully demonstrates their excellent scour resistance. In contrast, obvious fiber-melting characteristics are displayed in QF/PF and MF/PF. Part of the fiber surfacesares covered with ceramic fusion cladding, and fiber breakage and pulling phenomena are visible (Figure 11a). It is revealed that under case1 with lower airflow flushing, a more complete ceramic fused cladding layer is generated on the surface of QF/PF and MF/PF. The protective layer can effectively block the erosion of the carbon layer tissue from the high-temperature oxidative environment. Under the case4 of strong airflow erosion, the coverage of the fused cladding layer is significantly reduced. The reduction results in a large area of exposed carbon layer tissue and a marked increase in the probability of fiber melting. Notably, the surface of CF/PF exhibits significant ablation holes, a feature stemming primarily from two factors. Firstly, a notable difference exists in the coefficient of thermal expansion between carbon fibers and the resin matrix. Under high-temperature gradients, large thermal stresses are generated, leading to interface debonding. Secondly, carbon fibers lack a molten phase to bridge them to the matrix, which prevents the formation of an effective buffer layer. The combination of these two factors makes it easier for the hot gas flow to invade the interior of the material, which in turn leads to the formation of ablation holes.

### 2.5. Elemental Analysis of Ablative Layers

**Figure 12:**
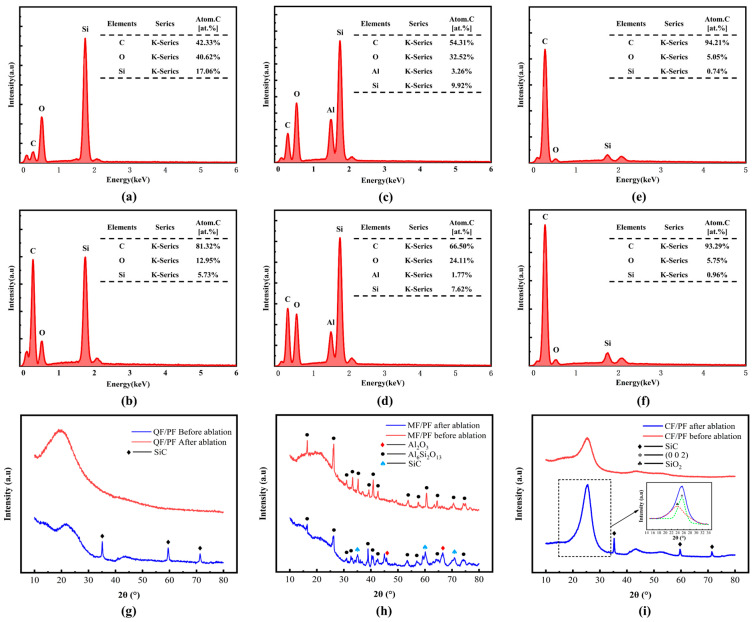
Elemental analysis of ablated surfaces and XRD spectrum before and after ablation. (**a**) QF/PF in case1. (**b**) QF/PF in case4. (**c**) MF/PF in case1. (**d**) MF/PF in case4. (**e**) CF/PF in case1. (**f**) CF/PF in case4. (**g**) QF/PF. (**h**) MF/PF. (**i**) CF/PF.

The chemical characteristics of the ablated surfaces of the three fiber-reinforced composites are revealed in Figure 12 through elemental examination and XRD comparison. Energy spectrum analysis reveals the presence of three elements, C, O, and Si, on the surface of all materials. Carbon accounts for the largest proportion, with its content in CF/PF exceeding 90%. It is revealed in a comparison of different cases that the carbon content under case1 is significantly lower than that under case4. The strong airflow scouring causes a large loss of the molten ceramic phase, reducing the surface ceramic coverage. The reduction is consistent with the decrease in the fused layer observed in the ablation morphology in Figure 9. Particularly noteworthy is that the Si content on the surface of CF/PF is less than 1%. The ablated surface is thus barely coated with ceramic oxide tissue, and the original carbon fiber skeleton and pyrolytic carbon layer are mainly retained. In contrast, Al is detected in MF/PF. It mainly originates from the Al_2_O_3_-SiO_2_ composite ceramic phase generated through the decomposition of its fiber component (3Al_2_O_3_-2SiO_2_) at high temperatures.

The characteristics of the Al_6_Si_2_O_13_ crystalline phase structure are revealed by XRD analysis. These characteristics are exhibited only in MF/PF among the three fiber-reinforced composites before ablation. After ablation, the generation of SiO_2_ and SiC ceramic phases are detected on the surface of all three composites. Among them, the source of SiO_2_ mainly involves the high-temperature melting of quartz and mullite fibers and the oxidation reaction of organic Si in the resin. During the ablation process, the overflow of pyrolysis gas leads to the formation of a boundary layer, which in turn creates a high-temperature anaerobic environment. Under this environment, SiO_2_ reacts with the activated carbon in the carbon layer to generate SiC. The specific reaction mechanism is shown in Equation (1). However, when oxygen intrudes, SiC exposed to an oxygen atmosphere undergoes oxidation reactions. In the temperature interval of 900–1600 °C, SiC experiences an active oxidation reaction to produce SiO, CO, and CO_2_ gases. The specific reaction process is described in Equations (2) and (3). In a high-temperature and low-oxygen environment, SiC then undergoes a passivated oxidation reaction to produce solid SiO_2_ and gaseous CO_2_, as shown in Equation (4). In addition, SiC experiences a crystalline transformation when the temperature climbs up to 2200 °C, the specific reaction is shown in Equation (5). Through the above series of complex chemical reactions, a ceramic fused cladding layer containing both SiO_2_ and SiC is finally formed on the surface of the ablative layer. It is worth noting that SiO_2_ is demonstrated to exist in an amorphous form in the XRD spectrum, corresponding to the large plate peak in the 15–35 ° interval. The peak is mainly composed of the hexagonal graphite structure (0 0 2) together with the amorphous SiO_2_, as can be seen in Figure 12i. Further analysis of the mullite fibers after ablation reveals the appearance of two crystalline phases, Al_2_O_3_ and Al_6_Si_2_O_13_. Among them, Al_6_Si_2_O_13_ is the melt product of mullite fibers at high temperature. Al_2_O_3_, on the other hand, is generated as mullite fibers undergo a high-temperature decomposition reaction, as described in Equation (6). The intensity of the characteristic diffraction peaks of Al_6_Si_2_O_13_ before ablation is significantly higher than that after ablation. It is indicated that the decomposition reaction of Al_6_Si_2_O_13_ occurs during high-temperature ablation, with the crystalline phase content reduced accordingly. An effective protective barrier is constructed on the surface of the ablative layer through the synergy of these two crystalline phases with SiO_2_ and SiC. The protective barrier can significantly isolate the erosion of hot air flow, and thus, the high temperature resistance of the composite material is enhanced accordingly.

(1) $${\mathrm{SiO}}_{2}\left(s\right)\;+\;3C\left(s\right)\to \mathrm{SiC}\left(s\right)\;+\;2\mathrm{CO}\left(g\right)$$

(2) $$\mathrm{SiC}\left(s\right)\;+\;O_{2}\left(g\right)\to \mathrm{SiO}\left(g\right)\;+\;\mathrm{CO}\left(g\right)$$

(3) $$\mathrm{SiC}\left(s\right)\;+\;3/2O_{2}\left(g\right)\to \mathrm{SiO}\left(g\right)\;+\;{\mathrm{CO}}_{2}\left(g\right)$$

(4) $$\mathrm{SiC}\left(s\right)\;+\;2O_{2}\left(g\right)\to {\mathrm{SiO}}_{2}\left(s\right)\;+\;{\mathrm{CO}}_{2}\left(g\right)$$

(5) 6H-SiC→3C-SiC

(6) $$3{\mathrm{Al}}_{2}O_{3}\cdot 2{\mathrm{SiO}}_{2}\left(s\right)\to {3{\mathrm{Al}}_{2}O_{3}\left(s\right)\;+\;\mathrm{SiO}}_{2}\left(s\right)$$

### 2.6. Ablation Rate

**Figure 13:**
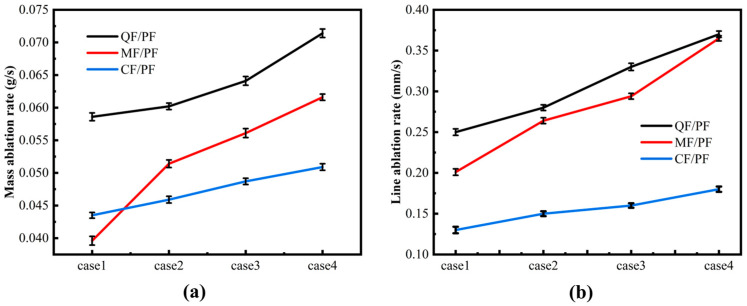
(**a**) Mass ablation rate. (**b**) Linear ablation rate.

A systematic study was conducted on three fiber-reinforced composites in an oxyacetylene ablation environment. The pattern of change in their properties under extreme thermal loading is revealed. With the increase in oxygen and acetylene flow rate, the ablation rates of all three materials exhibit an increasing trend, though a significant difference is observed (as shown in Figure 13). Under all four cases, there are the largest mass ablation rate and linear ablation rate for QF/PF. MF/PF follows, while the smallest ablation rate is seen in CF/PF. The mass ablation rate and linear ablation rate of QF/PF rises by 30.4% and 72% from case1 to case4, respectively. Typical exponential growth characteristics are shown in the growth curves of these rates. A sharp increase is noted from case3 to case4. This is mainly attributed to the melting and loss of the SiO_2_-SiC ceramic layer under high-speed gas flow, which leads to a marked decrease in the material’s resistance to ablative shear. The mass ablation rate and linear ablation rate of MF/PF are increased by 43.9% and 89.1% from case1 to case4, respectively. The growth trend exhibits a “Z” shape with notable jumps observed in case1 to case2 and case3 to case4, and it can be seen that the ablation rate of MF/PF varies with flow rate in a complex situation. In contrast, the best stability is demonstrated in CF/PF. The rise in mass and linear ablation rate from case1 to case4 is only 17.0% and 38.5%, respectively. This is mainly attributed to the ability of the carbon fiber skeleton to maintain its structural integrity at high temperatures. XRD and EDS analyses (shown in Figure 12) reveal that only two ceramic layers, SiO_2_ and SiC, are formed for QF/PF, whereas a four-phase composite ceramic layer consisting of Al_2_O_3_-Al_6_Si_2_O_13_-SiO_2_-SiC is generated for MF/PF. The ablation resistance is significantly enhanced with the presence of Al-based ceramic phases through several mechanisms. First, the high melting point and thermal stability of Al_2_O_3_ keep it stable at high temperatures. Second, the composite ceramic layer generated from Al_2_O_3_ and SiO_2_ effectively prevents heat conduction. Third, the higher viscosity of the molten Al_2_O_3_-SiO_2_ eutectic phase strengthens its ablation and erosion resistance. These properties make MF/PF superior to QF/PF in terms of mass ablation rate and linear ablation rate.

Under the oxyacetylene ablation condition with an oxygen flow rate of 950 L/h and an acetylene flow rate of 700 L/h, the linear ablation rate of the CF/PF composite reaches 1.3 mm/s. To further clarify its ablation resistance level, relevant literature data with similar test conditions are selected for comparative analysis: A linear ablation rate of 0.117 mm/s for the developed material is reported by Wang et al. [46] under the conditions of an oxygen flow rate of 400 L/h and an acetylene flow rate of 500 L/h. It is found by Yin et al. [47] that the linear ablation rate of the CBCF/PR composite is 0.119 mm/s in the ablation environment with the same oxygen flow rate (400 L/h) and acetylene flow rate (500 L/h). It should be noted that the oxyacetylene flow rates in the above-mentioned studies are lower, corresponding to a relatively mild ablation environment; thus, the linear ablation rates of their materials are generally lower than those in this study. However, overall, favorable ablation resistance performance is still exhibited by the CF/PF composite.

### 2.7. Ablation Mechanism

**Figure 14:**
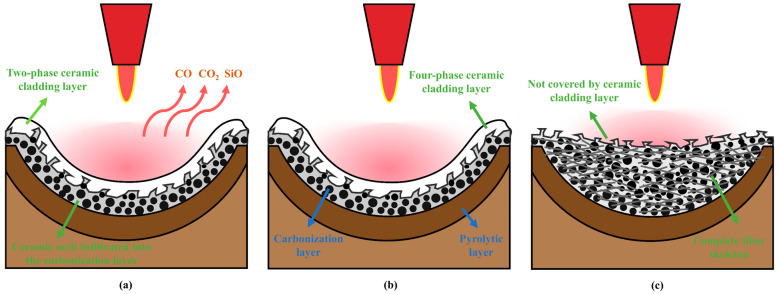
(**a**) Ablation mechanism of QF/PF. (**b**) Ablation mechanism of MF/PF. (**c**) Ablation mechanism of CF/PF.

The comparison diagram of ablation mechanisms for the three fiber-reinforced phenolic aerogel composites is shown in Figure 14. Owing to the differences in types of reinforcing fibers, distinct ablation mechanisms and performance characteristics are exhibited by the three composites during the ablation process. Variations in the intrinsic properties of fibers, such as differences in melting temperature and disparities in ceramic oxidation products formed after ablation, all exert an impact on the overall ablation performance of the composites.

High-temperature ablation and erosion are synergistically resisted in QF/PF through the in situ formation mechanism of the “SiO_2_-SiC two-phase ceramic cladding layer” combined with the excellent ablation resistance of the composite itself. At relatively low ablation temperatures, the resin matrix undergoes pyrolysis, releasing gaseous products including CO, CO_2_, and benzene ring derivatives. The large amount of heat is carried away through the escape of these gases, which functions as a heat dissipation effect. Meanwhile, the gas-phase barrier is formed on the material surface, and further penetration of high-temperature gas flow into the material interior is effectively prevented. When the temperature rises above 1700 °C, quartz fibers melt and absorb heat, and SiO_2_ and SiC ceramic cladding layers are generated under high-temperature oxygen-rich and oxygen-deficient environments, respectively. A portion of the molten material is scoured away by high-speed gas flow, while the other portion penetrates into the composite interior. Through this process, pores in the carbonized layer and pyrolysis layer are filled, the fractured carbon layer structure is bridged, and the material’s erosion resistance is enhanced. Under low heat flux conditions, this ceramic layer can form a continuous and stable protective film, exerting excellent thermal insulation and protective effects. However, under intense scouring by high heat flux, the liquid ceramic phase is prone to loss, leading to exposure of local carbon layers, accelerated inward diffusion of hot gas flow, increased risk of fiber melting, weakened overall protective effect, and thus a decline in ablation resistance.

MF/PF exhibits superior ablation resistance to QF/PF due to the “synergistic protection of multiphase ceramic layers”. The melting point of mullite fiber (1850 °C) is higher than that of quartz fiber. When quartz fiber melts and loses its reinforcing effect, mullite fiber still maintains a supporting role, effectively connecting the carbon layer structure and resisting the shear action of high-temperature gas flow. When the temperature exceeds 1850 °C, mullite fiber melts and absorbs heat, taking in part of the heat. Simultaneously, the Al_2_O_3_-Al_6_Si_2_O_13_-SiO_2_-SiC four-phase composite ceramic layer is formed. Among these phases, the high-temperature stability and ablation resistance of the material surface are improved by the Al-based ceramic phases, with the protective performance of MF/PF in extreme thermal environments further enhanced. With “intact skeleton support” as its core mechanism, the optimal ablation resistance stability is exhibited by CF/PF. In the ablation environment of 2200 °C, carbon fibers neither melt nor sublime, and the intact skeleton structure is always maintained. Continuous mechanical support is provided for the material, and structural collapse is avoided. In the early stage of ablation, the carbon layer formed by resin pyrolysis is tightly combined with the carbon fiber skeleton, constructing a relatively dense structure. However, the coverage of the surface ceramic layer generated after the ablation of organosilicon-modified resin is relatively low. Therefore, the ablation resistance of CF/PF mainly relies on the physical support provided through the carbon fiber skeleton. Due to the absence of fiber melting and loss, and the fact that the shear erosion of the carbon layer by high-speed gas flow can be effectively inhibited by the intact skeleton structure, an extremely low ablation rate can still be maintained by CF/PF even under high heat flux conditions. It is significantly superior to the other two materials, with the most excellent ablation resistance demonstrated.

## 3. Conclusions

In this study, phenolic aerogel composites reinforced with quartz fibers, mullite fibers, and carbon fibers are prepared using the RTM process. The mechanism governing how fiber types influence the microstructure, thermal properties, mechanical properties, and ablation-resistant behaviors of the materials is systematically revealed.

(1) The experimental results show that the resin matrix dominates the pore formation process of the composites. A highly homogeneous nanoporous structure is presented in all three fiber-reinforced systems, with an average pore size of 68.44–75 nm and a porosity of about 65%. It is demonstrated through thermogravimetric tests that the thermal decomposition behavior of the materials is affected by the fiber type. The fastest pyrolysis rate of the resin matrix is exhibited in CF/PF, due to the higher thermal conductivity of the composites. At 800 °C, the residual carbon rate of CF/PF is the lowest among the three fiber-reinforced composites. Lower thermal conductivity, higher specific heat capacity, and excellent thermal stability are possessed in QF/PF and MF/PF. In application scenarios requiring excellent thermal insulation, these systems show significant advantages due to such properties.
(2) The best mechanical properties are displayed in CF/PF. At 25 °C, its tensile strength and bending strength are 54.62 MPa and 29.69 MPa, respectively, which are 31.1% and 527.1% higher than those of QF/PF and 8.32% and 17.35% higher than those of MF/PF, respectively. This performance is mainly attributed to the high specific strength and modulus of the carbon fibers, along with the good interfacial bonding ability between the fibers and the matrix. At 200 °C, CF/PF still maintains the best mechanical properties, with its tensile strength and bending strength at high temperature being 41.6 MPa and 29.03 MPa, respectively, which are 33.6% and 13.93% higher than those of QF/PF and 466.8% and 15.75% higher than those of MF/PF, respectively. At this temperature, the bending property retention ability of CF/PF is notably better than those of QF/PF and MF/PF. Their complete fiber backbone network effectively compensates for the loss of strength caused by matrix pyrolysis. Comprehensive mechanical property evaluation indicates that CF/PF is most suitable for applications involving high mechanical loads on heat-insulating components.
(3) At high temperatures, the SiO_2_-SiC ceramic layer formed in QF/PF shows good protection under low heat flow conditions (case1). Under high heat flow conditions (case4), however, the ablation resistance of QF/PF decreases due to the loss of the ceramic layer, and the linear ablation rate increases significantly. The high-viscosity Al_2_O_3_-Al_6_Si_2_O_13_-SiO_2_ multiphase melt is produced when MF/PF ablates. Superior ablation resistance is demonstrated in this multiphase melt compared to QF/PF. The most outstanding ablation resistance is found in CF/PF. Its line and mass ablation rates are significantly lower than those of QF/PF and MF/PF, owing to the high temperature stability of the carbon fibers and the mechanical support afforded by the intact skeleton. Combined ablation data indicate that CF/PF exhibits optimal ablation resistance in extreme thermal environments.

## 4. Materials and Methods

### 4.1. Materials

In this study, the thermoplastic phenolic resin from Wuhan Yingcheng Lifa Chemical Co., Ltd. (Wuhan, China) is used as the matrix material. Its density is 0.97 g/cm^3^, viscosity is 19 mPa·s, solid content is 36%, and solvent is ethanol. To improve the performance of the resin, methyltriethoxysilane is used for organosilicon modification. Hexamethylenetetramine from the same manufacturer is used as the curing agent. The reinforcing materials are three kinds of fiber knitted felts (quartz fiber felts, mullite fiber felts and carbon fiber felts) from Wuhan Huan Yu Fei Long New Material Technology Co., Ltd. (Wuhan, China). The knitted felts are prepared through the process of alternating stacking of woven fabric and fiber felts, with a bulk density of 0.18 g/cm^3^.

### 4.2. Preparation of Composites

Phenolic aerogel composites are prepared using the RTM process [48]. Firstly, the thermoplastic phenolic resin and the curing agent hexamethylenetetramine were weighed at a mass ratio of 4:1. The mixture was then stirred on a magnetic stirrer at 500 rpm until completely homogeneous. Subsequently, the homogeneously mixed resin system was injected into molds filled with fiber preforms. After resin injection was completed under 0.3 MPa nitrogen pressure to ensure complete infiltration of the resin into the fiber preforms, the molds were placed in an oven at 100 °C for 20 h to complete the sol–gel process of the phenolic resin solution, resulting in wet gels. This was followed by drying at 60 °C for 24 h under ambient pressure to completely remove water and by-products such as ethanol produced from the polycondensation reaction, ultimately yielding the aerogel composites.

### 4.3. Characterization

The three fiber-reinforced phenolic aerogel composites were tested, including mechanical, thermal, and ablative property tests, as well as micro-morphological characterization and elemental analysis. Mechanical performance tests were carried out using the electronic universal testing machine (CMT6103, Meters Industrial Systems, Eden Prairie, MN, USA), with five valid samples set for each group of tests. Tensile and bending performance tests were conducted at 25 °C and 200 °C, respectively. The loading rates were 5 mm/min for tension and 2 mm/min for bending. Thermal conductivity in the range of 20–200 °C was determined using a thermal conductivity meter (TPS 2500S, Hot Disk, Gothenburg, Sweden), with three valid samples tested for the measurement. Thermogravimetric analysis (TGA) was performed on three valid sample groups with a comprehensive thermal analyser (Thermo plus EVO2 DSCvesta, Rigaku, Shibuya-ku, Tokyo, Japan) under test conditions of a 50 mL/min heat flow rate, a 25–800 °C temperature range, and a 10 °C/min temperature increase rate. Specific heat capacity was measured using a medium-temperature-specific heat capacity tester (STA 449F3, NETZSCH, Selb, Germany), with three valid samples set for each group of tests. The coefficient of thermal expansion in the range of 20–150 °C was measured with a thermo-mechanical analyser (TMA 402 F3, NETZSCH, Selb, Germany), with three valid samples included in each group of tests. Physical property tests included the following: the density of the composites was tested using a density tester (AccuPyc 1340; Micromeritics, Norcross, GA, USA), with five valid samples set for each group of tests. The porosity and average pore size of the materials were tested using the mercury injection instrument (MI, Autopore IV 9510, Micromeritics Instrument Co., Norcross, GA, USA), with three valid samples included in each group of tests. Ablation resistance properties were evaluated using an oxyacetylene ablation tester (HDQCS-N, Xi’an HanDa Measurement And Control Technology Co., Ltd., Xi’an, China), with five valid samples included in each group of tests, with the ablation time of 10 s. Micro-morphological characterization was carried out using a scanning electron microscope (SEM, Zeiss Sigma 300, Oberkochen, Germany). Elemental analyses were performed using an X-ray diffractometer (XRD, Ultima IV diffractometer, Rigaku, Tokyo, Japan) with a scanning range of 10–80 ° and a scanning speed of 2 °/min.

## Figures and Tables

**Table 1:** Tensile stress, tensile modulus and elongation at break at 25 °C.

Typology	Tensile Stress (MPa)	Standard Deviation of Tensile Stress (MPa)	Tensile Modulus (GPa)	Standard Deviation of Tensile Modulus (GPa)
QF/PF	41.66	1.32	2.08	0.073
MF/PF	8.71	0.095	1.03	0.071
CF/PF	54.62	1.12	6.6	0.21

**Table 2:** Tensile stress, tensile modulus and elongation at break at 200 °C.

Typology	Tensile Stress (MPa)	Standard Deviation of Tensile Stress (MPa)	Tensile Modulus (GPa)	Standard Deviation of Tensile Modulus (GPa)
QF/PF	31.14	1.453	1.72	0.105
MF/PF	7.34	0.105	0.7	0.084
CF/PF	41.6	1.562	4.71	0.196

**Table 3:** Test items of ablation rate.

Case	Oxygen Flow Rate (L/h)	Acetylene Flow Rate (L/h)
Case1	950	700
Case2	1080	800
Case3	1200	900
Case4	1350	1000

## Data Availability

The data presented in this study are available on request from the corresponding author.
